# Intracellular hypoxia measured by ^18^F-fluoromisonidazole positron emission tomography has prognostic impact in patients with estrogen receptor-positive breast cancer

**DOI:** 10.1186/s13058-018-0970-6

**Published:** 2018-07-27

**Authors:** Aya Asano, Shigeto Ueda, Ichiei Kuji, Tomohiko Yamane, Hideki Takeuchi, Eiko Hirokawa, Ikuko Sugitani, Hiroko Shimada, Takahiro Hasebe, Akihiko Osaki, Toshiaki Saeki

**Affiliations:** 1 Department of Breast Oncology, Saitama Medical University, 38 Morohongo, Moroyama-machi, Irumagun, Saitama, 350-0451, Japan; 2 Department of Breast Oncology, Saitama Medical University International Medical Center, 1397-1 Yamane, Hidaka, Saitama, 350-1241, Japan; 3 Department of Nuclear Medicine, Saitama Medical University International Medical Center, 1397-1 Yamane, Hidaka, Saitama, 350-1241, Japan; 4 Department of Pathology, Saitama Medical University International Medical Center, 1397-1 Yamane, Hidaka, Saitama, 350-1241, Japan

**Keywords:** Breast cancer, Hypoxia, Prognosis, ^18^F-fluoromisonidazole, Positron emission tomography

## Abstract

**Background:**

Hypoxia is a key driver of cancer progression. We evaluated the prognostic impact of ^18^F-fluoromisonidazole (FMISO) prior to treatment in patients with breast cancer.

**Methods:**

Forty-four patients with stage II/III primary breast cancer underwent positron emission tomography/computed with ^18^F-fluorodeoxyglucose (FDG-PET/CT) and FMISO. After measurement by FDG-PET/CT, the tissue-to-blood ratio (TBR) was obtained using FMISO-PET/CT. FMISO-TBR was compared for correlation with clinicopathological factors, disease-free survival (DFS), and overall survival (OS). Multiplex cytokines were analyzed for the correlation of FMISO-TBR.

**Results:**

Tumors with higher nuclear grade and negativities of estrogen receptor (ER) and progesterone receptor had significantly higher FMISO-TBR than other tumors. Kaplan-Meier survival curves showed that patients with a higher FMISO-TBR (cutoff, 1.48) had a poorer prognosis of DFS (*p* = 0.0007) and OS (*p* = 0.04) than those with a lower FMISO-TBR. Multivariate analysis indicated that higher FMISO-TBR and ER negativity were independent predictors of shorter DFS (*p* = 0.01 and 0.03). Higher FMISO-TBR was associated with higher plasma levels of angiogenic hypoxic markers such as vascular endothelial growth factor, transforming growth factor-α, and interleukin 8.

**Conclusions:**

FMISO-PET/CT is useful for assessing the prognosis of patients with breast cancer, but it should be stratified by ER status.

**Trial registration:**

UMIN Clinical Trials Registry, UMIN000006802. Registered on 1 December 2011.

**Electronic supplementary material:**

The online version of this article (10.1186/s13058-018-0970-6) contains supplementary material, which is available to authorized users.

## Background

There is robust evidence that tumor hypoxia, a distinguishing feature of various solid tumors, is a major contributor to cancer progression and resistance to therapy [1, 2]. The development of hypoxia in the tumor microenvironment is a dynamic process that is mediated primarily by hypoxia-inducible factor (HIF)-1 through the activation of unregulated glycolysis, angiogenesis, and p53 mutation [3]. However, despite wide understanding of the negative impact of tumor hypoxia associated with cancer progression, only few clinical tools are available to directly measure the hypoxic activity of breast cancer, which to date has not been stratified by intrinsic tumor subtypes by means of immunohistochemistry [4, 5]. Although the measurement of hypoxic activity by the methods of partial pressure of Oxygen electrode or molecular assessment using tissue samples has been reported to be useful, such methods are invasive and restricted to accessible tumors [6, 7]. A noninvasive method of measuring hypoxia is clinically required for application in metastatic cancer and for monitoring therapeutic response. Currently, ^18^F-fluoromisonidazole (FMISO) positron emission tomography (PET) with computed tomography (CT) is the most widely accepted imaging technology available for the localization and quantification of intracellular hypoxia of tumor in vivo [8]. FMISO taken in cells is reduced by nitroreductase enzymes under low partial pressure of oxygen. FMISO is not trapped in necrotic cells or normoxic cells, because no reduction by the enzymes or reoxidization of the tracer eventually diffuses out through vessels. Therefore, FMISO selectively accumulates in viable hypoxic cells but not in necrotic cells and normoxic cells [9]. Chapman et al. indicated that nitroimidazole has an affinity for hypoxic cells, suggesting that it may be useful for extracting images of hypoxic cells [10].

The utility of FMISO-PET/CT in oncology has been well documented in several clinical studies in patients with gliomas, non-small lung cancer, head and neck cancer, and breast cancer [11–14]. The general consensus derived from these studies is that FMISO-PET/CT provides heterogeneity of hypoxic activity depending on cancer types and promises to help facilitate image-guided therapy [15]. Although previous reports on various solid tumors have shown a worse prognosis if patients had higher tumor uptake of FMISO, there are no reports on patients with primary breast cancer.

We previously demonstrated in the clinical imaging study that when patients with advanced breast cancer underwent serial FMISO-PET/CT scans at pretherapy and after two cycles of bevacizumab and paclitaxel, a high FMISO uptake of tumor was characteristics of nonresponders to bevacizumab combined chemotherapy [16]. We conducted the present clinical study to determine whether the degree of FMISO uptake predicts survival in patients with primary breast cancer. The primary aim was to evaluate the association of baseline hypoxia as measured by FMISO-PET/CT prior to the treatment of primary breast cancer with disease-free survival (DFS). Multiplex cytokine analysis was also conducted.

## Methods

### Patient enrollment

Between December 2011 and March 2015, a total of 44 patients with clinical stage II/III pathologically proven invasive breast cancer of the primary site were enrolled. Patients who had already received prior treatments for the disease before PET scanning were excluded. Other exclusion criteria included patients with pregnancy, those aged < 18 years, and those with bilateral breast cancer. Eligible patients underwent ^18^F-fluorodeoxyglucose (FDG)-PET/CT first and then FMISO-PET/CT separately several days after FDG-PET/CT. They underwent magnetic resonance imaging (MRI) of the breasts to measure tumor size and the spread of tumor invasion. PET was performed at least 1 week after core biopsy. The pathological diagnosis was determined by agreement between two pathologists, including one experienced breast pathologist (TH). Eleven patients primarily received definitive surgery. Thirty-three patients received neoadjuvant chemotherapy and then underwent definitive surgery. They received adjuvant treatment with endocrine therapy and/or radiotherapy if required according to practice guidelines. The ethics committee of the Saitama Medical University International Medical Center approved the study. Written informed consent was obtained from each patient who participated in the study. The study was registered in the UMIN Clinical Trials Registry (UMIN000006802).

### PET study protocol

The Biograph 6/16 scanner (Siemens, Erlangen, Germany) was used for FDG-PET/CT and FMISO-PET/CT. The patients fasted for at least 6 hours before the administration of tracers. The static PET images were acquired at 1 and 2 hours after the intravenous injections of FDG (3.7 MBq/kg) and FMISO (7.4 MBq/kg), respectively. The images were acquired using the three-dimensional mode and reconstructed with ordered subset expectation maximization. On the FMISO-PET/CT study, two nuclear medicine physicians (TY, IK) determined a tumor lesion of the target by referring to the FDG-PET/CT and MRI images. ROIs were placed over the primary tumor, and maximum standardized uptake value (SUV_max_) was measured. SUV was calculated according to the following formula: SUV = activity concentration in ROI (MBq/ml)/injection dose (MBq/kg body weight). In addition, quantification of FMISO in image-derived tissue regions used as surrogates for blood activity was performed. The SUV of 2-cm circular regions placed on approximately 2 cm of axial distance over the left ventricular cavity was measured. The tissue-to-blood ratio (TBR) was calculated to normalize to the reference activity [17].

### Immunohistochemistry

The expression of estrogen receptor (ER), progesterone receptor (PgR), human epidermal growth factor receptor 2 (HER2), and MIB1 was immunohistochemically examined for all specimens. The data were obtained from pathological reports. In the present study, a hormone receptor status of ≥ 10% nuclear staining was regarded as positive, whereas any status of < 10% nuclear staining was regarded as negative. Cases with a score of > 3 were considered to overexpress HER2. If the score was > 2, fluorescence in situ hybridization (FISH) was performed. When the amplification of *HER2* was detected using FISH, the result was considered to be positive. Others were considered as negative. We set the cutoff at 14% of Ki-67 labeling index for differentiating breast tumors into luminal A type and luminal B type [18].

### Multiplex cytokine assay

Seventeen patients agreed to participate in the blood biomarker study and peripheral blood samples of the patients were collected for measuring cancer-related cytokines. Plasma samples were separated by centrifugation, aliquoted, and stored at − 80 °C. Enzyme-linked immunosorbent assays were performed for epidermal growth factor, eotaxin, fibroblast growth factor 2, Flt3L, fractalkine, granulocyte colony-stimulating factor, granulocyte-macrophage colony-stimulating factor, GRO, interferon (IFN)-α2, IFN-γ, interleukin (IL)-10, IL-12p40, IL-12p70, IL-13, IL-15, IL-17A, IL-1α, IL-1β, IL-1RA, IL-2, IL-3, IL-4, IL-5, IL-6, IL-7, IL-8, IL-9, IL-10, monocyte chemoattractant protein (MCP)-1, MCP-3, macrophage-derived chemokine, macrophage inflammatory protein (MIP)-1α, MIP-1β, sCD40L, transforming growth factor (TGF)-α, tumor necrosis factor-β, and vascular endothelial growth factor (VEGF) with a chemiluminescence immunoassay-certified multiplex protein array using the MILLIPLEX MAP Human Cytokine/Chemokine Magnetic Bead Panel (HCYTMAG-60 K-PX38; EMD Merck Millipore, Darmstadt, Germany). All samples were assayed in duplicates.

### Statistics

The primary objective of the study was to determine the association of baseline FMISO-TBR with DFS in patients with newly diagnosed primary breast cancer. Secondary aims included correlation of FMISO-TBR with tumor characteristics and overall survival (OS). The correlations between parameters were assessed using Student’s *t* test. All data are presented as mean ± SD. Kaplan-Meier survival estimates for time to recurrence and death were generated along with the median survival time with its 95% CI. The log-rank test was performed for comparisons between the survival curves. ROC curves for parameters constructed for DFS and AUC were estimated. The tentative cutoff value of FMISO-TBR was set as 1.48, referencing previous study results [19]. Prognostic parameters were separately modeled using a univariate Cox proportional hazards regression model for DFS and OS. The HR, along with its 95% CI and *p* value based on the Wald statistic, was reported. A *p* value < 0.05 was considered statistically significant. All analyses were performed using Medcalc version 17.6 statistical software (Medcalc, Ostend, Belgium).

## Results

### Patient characteristics

Forty-four patients with newly diagnosed primary breast cancer underwent both FDG-PET/CT and FMISO-PET/CT scans. All patients underwent definitive surgery (mastectomy or partial resection) with sentinel lymph node biopsy and/or axillary dissection. Twenty-two (50%) patients had clinical stage II breast cancer, and the others had clinical stage III breast cancer. Patient characteristics, including tumor size, histology, and hormone receptor and HER2 status, are shown in Table 1. Overall, 39 (88.6%) patients received four to eight courses of standard chemotherapy including anthracycline and/or taxane in the adjuvant or neoadjuvant setting. All patients with hormone receptor-positive breast cancer received adjuvant endocrine therapy including tamoxifen or an aromatase inhibitor for at least 5 years.

**Table 1:** Patient characteristics and comparison of tumor uptake of fludeoxyglucose/^18^F-fluoromisonidazole

				FDG-SUV_max_			FMISO-TBR		
Variables		Number	(%)	Mean	SD	*p* Value	Mean	SD	*p* Value
Number of patients		44							
Age, years	Average	53.5							
	(range)	(38–78)							
	< 50	20	(45.4)	11.1	4.4	0.02	1.3	0.4	0.3
	≥ 50	24	(45.5)	7.9	4.2		1.2	0.4	
Tumor size	Average	40.9							
	(range)	(14–80)							
	< 4 cm	25	(56.8)	8	3.6	0.02	1.1	0.4	0.03
	≥ 4 cm	19	(43.1)	11.1	5.1		1.4	0.4	
Histology	IDC	41	(93.1)	9.6	4.4	0.1	1.2	0.4	0.9
	Others	3	(6.8)	6	3.5		1.2	0.7	
Nuclear grade	1/2	17	(38.6)	7.4	3.6	0.06	1	0.2	0.007
	3	21	(47.7)	10	4.7		1.4	0.5	
	Unknown	6	(13.6)	–			–	–	
Nodal involvement	0	12	(27.2)	6.8	3.3	0.01	1.2	0.4	0.9
	≥ 1	32	(72.7)	10.5	4.6		1.2	0.4	
ER	+	26	(59)	9.1	4.2	0.7	1.1	0.3	0.04
	–	18	(40.9)	9.6	5.1		1.4	0.5	
PgR	+	19	(43.1)	7.9	3.1	0.07	1	0.2	0.009
	–	25	(56.8)	10.4	5.2		1.4	0.5	
HER2	+	7	(15.9)	7.8	3.6	0.3	1.2	0.2	1
	–	37	(84)	9.6	4.7		1.2	0.4	
Luminal A		7	(15.9)	7.8	2.5	0.4	0.9	0.2	0.03
Luminal B		16	(36.4)	9.7	4.8		1.1	0.3	
TNBC		14	(31.8)	10.5	5.4		1.4	0.5	
HER2		7	(15.9)	7.9	3.6		1.2	0.2	
Chemotherapy	+	39	(88.6)	9.7	4.6	0.1	1.2	0.4	0.01
	–	5	(11.4)	6.4	3		0.7	0.1	
Endocrine therapy	+	26	(59)	9.1	4.2	0.7	1.1	0.3	0.04
	–	18	(40.9)	9.6	5.1		1.4	0.5	

*Abbreviations: FDG* ^18^F-fluorodeoxyglucose, *FMISO* ^18^F-fluoromisonidazole, *IDC* Invasive ductal carcinoma, *ER* Estrogen receptor, *PgR* Progesterone receptor, *TBR* Tissue-to-blood ratio, *TNBC* Triple-negative breast cancer, *HER2* Human epidermal growth factor receptor 2, *SUV*_*max*_ Maximum standardized uptake value

### FDG-PET and FMISO-PET imaging markers

**Fig. 1:**
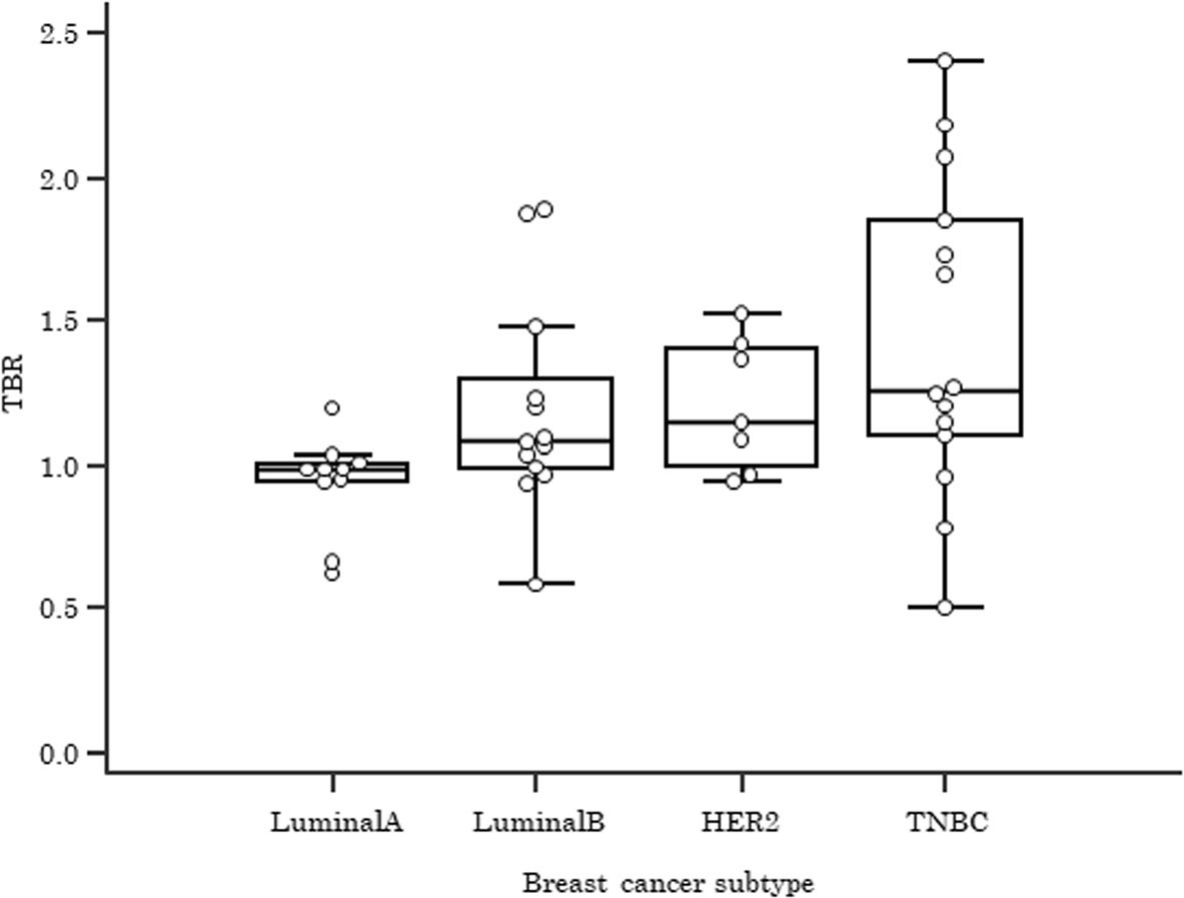
Tumor ^18^F-fluoromisonidazole uptake and intrinsic subtypes. *TBR* Tissue-to-blood ratio, *HER2* Human epidermal growth factor receptor 2, *TNBC* Triple-negative breast cancer

As shown in Table 1, FDG-SUV_max_ was significantly correlated with age (*p* = 0.02), tumor size (*p* = 0.02), and nodal metastasis (*p* = 0.01), and FMISO-TBR was significantly correlated with larger tumor size (*p* = 0.03), higher nuclear grade (*p* = 0.007), and negativities of estrogen receptors (*p* = 0.04) and progesterone receptor (*p* = 0.009). When FDG-SUV_max_ and FMISO-TBR were compared, the correlations between the pairs of tracers in 44 patients were statistically significant (*r* = 0.66; 95% CI, 0.48–0.80; *p* < 0.0001). Regarding intrinsic subtypes, as shown in Fig. 1, triple-negative breast cancer tumors had a significantly higher FMISO-TBR than luminal A tumors (*p* = 0.03). There were no significant differences between luminal A and luminal B tumors (*p* = 0.1) or between luminal A and HER2-type tumors (*p* = 0.1).

### Patient prognosis

**Fig. 2:**
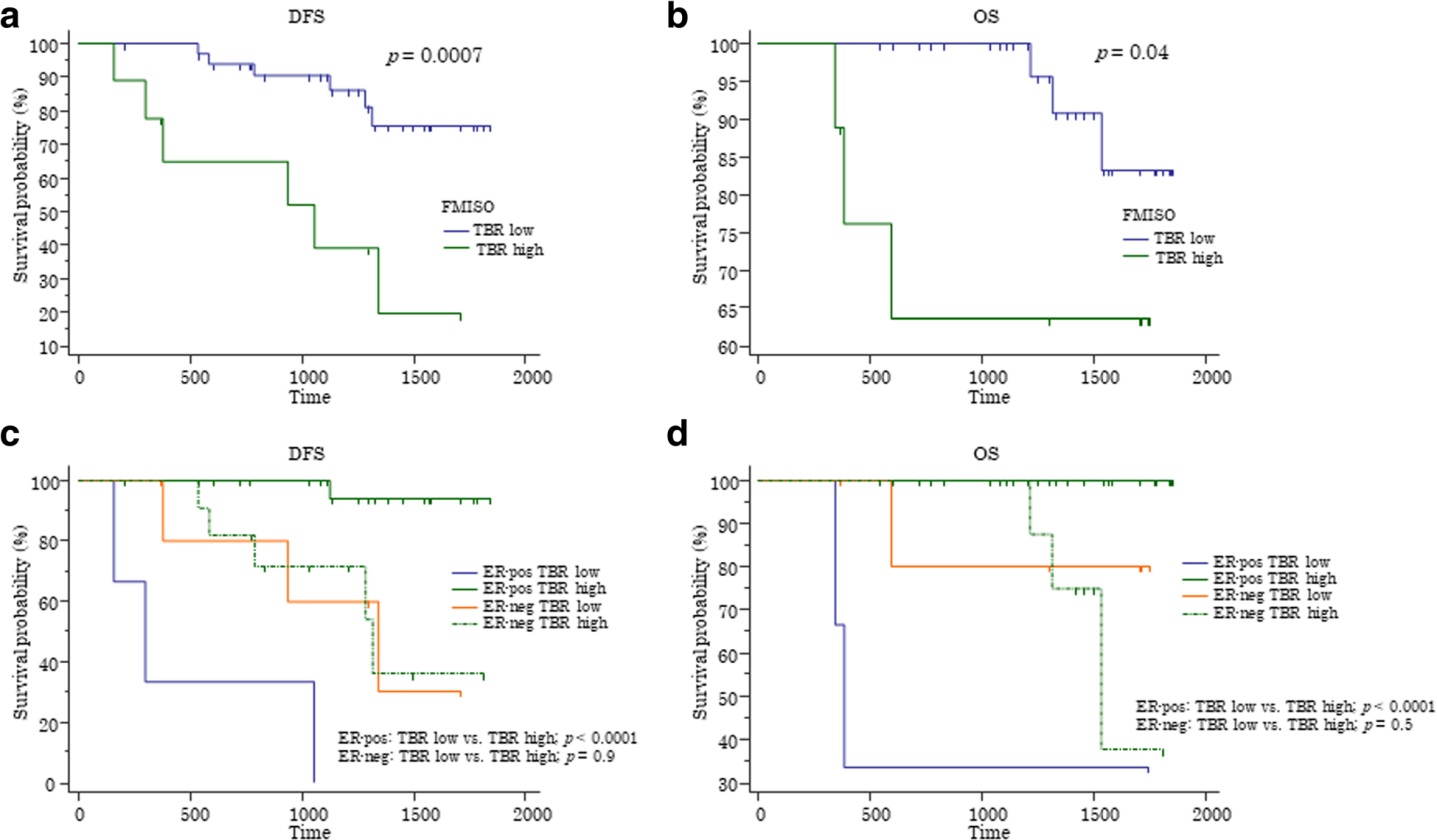
Survival curves. **a** Disease-free survival (DFS). **b** Overall survival (OS). **c** DFS stratified by estrogen receptor (ER) status. **d** OS stratified by ER status. The tentative cutoff value of 1.48 separates tumors with higher ^18^F-fluoromisonidazole tissue-to-blood ratio (TBR high) from those with lower ^18^F-fluoromisonidazole tissue-to-blood ratio (TBR low)

The median time of DFS and OS for the 44 patients were 1173 days (95% CI, 980–1274 days) and 1355 days (95% CI, 1208–1529 days), respectively. ROC analysis revealed that FMISO-TBR (AUC = 0.71; 0.1 SE; 95% CI, 0.56–0.84) was more predictive of DFS than was FDG-SUV_max_ (AUC = 0.55; 0.11 SE; 95% CI, 0.35–0.66; *p* = 0.003). When the tentative cutoff value of FMISO-TBR was set as 1.48, Kaplan-Meier survival analysis showed that tumors with higher FMISO-TBR had a poorer prognosis than those with lower FMISO-TBR with statistical significance for DFS (*p* = 0.0007) (Fig. 2a) and OS (*p* = 0.04) (Fig. 2b). When stratified by ER status, the results were found to be unique. FMISO-TBR in ER-negative tumors had no prognostic impact for DFS (*p* = 0.9) or OS (*p* = 0.5), whereas ER-positive tumors with higher FMISO-TBR had a poorer prognosis than those with lower FMISO-TBR with statistical significance for DFS (*p* < 0.0001) (Fig. 2c) and for OS (*p* < 0.0001) (Fig. 2d).

### Cox regression analysis

In the univariate Cox model, higher FMISO-TBR (*p* = 0.002) and ER negativity (*p* = 0.03) were significantly associated with a shorter DFS, as shown in Table 2. Both factors maintained their significant associations in a multivariate Cox model that included tumor size, nuclear grade, and nodal involvement. None of the markers had significant associations with OS.

**Table 2:** Results of Cox regression analysis

Disease-free survival variables		Univariable analysis			Multivariable analysis		
		HR	(95% CI)	*p* Value	HR	(95% CI)	*p* Value
Tumor size	< 4 cm	1		0.6	1		0.2
	≥ 4 cm	1.35	(0.43 to 4.19)		0.43	(0.09 to 1.94)	
Nuclear grade	1/2	1		0.9	1		0.06
	3	1	(0.28 to 3.56)		0.15	(0.02 to 1.13)	
ER	+	1		0.03	1		0.03
	–	3.72	(1.12 to 12.3)		7.53	(1.17 to 48.33)	
Nodal involvement	–	1		0.09	1		0.4
	+	5.75	(0.74 to 44.2)		2.25	(0.25 to 19.9)	
FMISO-TBR	< 1.48	1		0.002	1		0.005
	≥ 1.48	5.72	(1.83 to 17.82)		11.35	(2.07 to 62.23)	
Chemotherapy		1		0.9	N/A		
	+	368,675.3	(5.3E-168 to 25.4E + 177)				
Overall survival variables		Univariable analysis			Multivariable analysis		
		HR	(95% CI)	*p* Value	HR	(95% CI)	*p* Value
Tumor size	< 4 cm	1		0.6	1		0.7
	≥ 4 cm	1.45	(0.29 to 7.15)		0.71	(0.1 to 4.71)	
Nuclear grade	1/2	1		0.9	1		0.2
	3	1.09	(1.18 to 6.57)		0.21	(0.01 to 2.82)	
ER	+	1		0.2	1		0.1
	–	3.03	(0.55 to 16.5)		10.24	(0.57 to 181.96)	
Nodal involvement	–	1		0.9	1		0.9
	+	411,595.95	(9.7E-202 to 1.74E + 210)		141,679.29	(7.17E-223 to 27.97E + 231)	
FMISO-TBR	< 1.48	1		0.06	1		0.1
	≥ 1.48	4.43	(0.88 to 22.17)		7.59	(0.63 to 90.68)	
Chemotherapy		1		0.9	N/A		
	+	122,960.7	(6.7E-167 to 222.6E + 174)				

*Abbreviations: FMISO* ^18^F-fluoromisonidazole, *ER* Estrogen receptor, *TBR* Tissue-to-blood ratio

### Multiplex cytokine analysis

Plasma samples were used for multiplex cytokine analysis. As shown in Table 3, tumors with higher FMISO-TBR had significantly higher plasma concentrations of VEGF (pg/ml) (272.2 ± 188.4 vs. 62.1 ± 49.5, *p* = 0.006), TGF-α (pg/ml) (5.3 ± 2.4 vs. 1.6 ± 2.0, *p* = 0.01), and IL-8 (pg/ml) (14.7 ± 8.9 vs. 4.8 ± 2.9, *p* = 0.01) than tumors with lower FMISO-TBR (Student’s *t* test).

**Table 3:** Results of multiplex cytokine analysis

	FMISO-TBR		
	< 1.48	≥ 1.48	
Factors	Mean ± SD	Mean ± SD	*p* Value
VEGF, pg/ml	62.1 ± 49.5	272.2 ± 188.4	0.006
TGF-α pg/ml	1.6 ± 2.0	5.3 ± 2.4	0.01
IL-8 pg/ml	4.8 ± 2.9	14.7 ± 8.9	0.01

*Abbreviations: VEGF* Vascular endothelial growth factor, *TGF-α* Transforming growth factor-α, *IL* Interleukin

## Discussion

We found that the activity of intracellular hypoxia, as measured by FMISO-PET/CT, was associated with a shorter DFS in patients with primary breast cancer. The results provide clinical evidence of the negative prognostic value of tumor hypoxia among patients with breast cancer. Our findings are in agreement with previously published data on patients with glioma [20, 21], head and neck cancer [11, 22, 23], and non-small cell lung cancer [24]. Moreover, the multivariate analysis in the present study indicated tumor hypoxia and ER status as independent from tumor size, nuclear grade, and nodal involvement with regard to the DFS of patients with primary breast cancer. ER-positive tumors showed a more prognostic impact on FMISO-TBR than ER-negative tumors. This finding suggests that although FMISO-TBR is an indicator of metastatic potential of breast tumors, its hypoxic activity should be stratified by ER status when assessing the baseline risk of prognosis. Our findings regarding hypoxia and ER status were in line with previous studies that used complementary DNA microarrays. For instance, a 13-gene hypoxia signature was at a much higher level in the basal-like and claudin-low groups than in the luminal groups, and the HER2-enriched group had a wide deviation of expression levels of hypoxia-related gene signatures [25]. Interestingly, although ER-negative tumors had more attribution to a higher level of FMISO-TBR than luminal A tumors, FMISO-TBR showed a more prognostic impact in ER-positive tumors than in ER-negative tumors. This may be because hypoxia drives angiogenesis, metastasis, and hyperglycolysis via the activation of phosphoinositide 3-kinase (PI3K)-AKT signaling, and ER-negative tumors have a higher hypoxic activity than ER-positive tumors. However, the prognostic relevance of ER-negative tumors may be independent of their hypoxic status. In contrast, because ER-positive tumors were regulated by both ER and PI3K-AKT signaling, the dominant activation of PI3K-AKT signaling compared with that of ER signaling in ER-positive tumors may have led to a higher hypoxic activity and poorer prognosis.

Multiplex cytokine analysis revealed a strong relationship of tumor FMISO-TBR and cytokines/chemokines such as VEGF, TGF-α, and IL-8. HIF-1α, a transcription factor, regulates the expression of numerous genes of potent angiogenic cytokines, such as VEGF and TGF-α. IL-8 is a proinflammatory chemokine that belongs to the CXC subfamily, which directly stimulates VEGF. A gene expression database using Kaplan-Meier plotter (http://kmplot.com/analysis) revealed that high levels of these three cytokines were associated with poor relapse-free survival of patients with breast cancer. Thus, hypoxia induced several cytokines and chemokines, which could provide a direct determinant for the metastatic potential in patients with breast cancer. The clinical application of hypoxia imaging has provided essential information on tumor microenvironmental biology, adding to information on tumor size, growth factor receptors, and cellular proliferation. Hypoxia imaging may enable tailoring therapeutic strategies based on baseline risk assessment [26–29].

Our study had certain limitations. Regarding the study protocol of FMISO-PET/CT, we considered a cutoff value of 1.48 of FMISO-TBR as a reasonable threshold by evaluating the results of the present study. There was a high correlation between FMISO-SUV_max_ and FMISO-TBR, as shown in Additional file 1: Figure S1; however, the use of SUV_max_ is not appropriate by itself, because clearance of background activity needs be accounted for. In the present study, we found a difference in FMISO uptake between ER-positive and ER-negative breast cancer. However, how ER is involved with an imaging biomarker for hypoxia remains uncertain. Because hypoxic parameters and the cutoff value are considered tentative, further investigation is required using numerous patients’ data for calculating an optimal cutoff value to identify patients with poor prognosis. Considering a limited number of patients and the single-center study design, further studies with a larger population in a multicenter trial will be required to validate our findings, because several studies have used different protocols of PET scanning and devices [13, 30, 31].

## Conclusions

To the best of our knowledge, this is the first report showing that pretherapy FMISO uptake in primary breast cancer is a strong predictor for survival among patients with ER-positive breast cancer but not among those with ER-negative breast cancer. FMISO-PET/CT noninvasively provides hypoxic information and helps identify patients with a baseline risk of early recurrence and those eligible for antiangiogenic therapy, regardless of tumor size, nuclear grade, and nodal metastasis. Further prospective studies using FMISO-PET/CT are warranted before a hypoxia imaging-directed therapy is developed.

## Additional file

Additional file 1: **Figure S1.** Correlation between tumor FMISO-SUV_max_ and FMISO-TBR. Regression plots between tumor FMISO-SUV_max_ and FMISO-TBR showed high correlation at *r* = 0.94. (TIF 66 kb)
