# Correction to: Intracellular hypoxia measured by F-18 fluoromisonidazole positron emission tomography has prognostic impact in patients with estrogen-receptor positive breast (BRCR-D17-00693)

**DOI:** 10.1186/s13058-018-1038-3

**Published:** 2018-09-05

**Authors:** Aya Asano, Shigeto Ueda, Ichiei Kuji, Tomohiko Yamane, Hideki Takeuchi, Eiko Hirokawa, Ikuko Sugitani, Hiroko Shimada, Takahiro Hasebe, Akihiko Osaki, Toshiaki Saeki

**Affiliations:** 1 Department of Breast Oncology, Saitama Medical University Hospital, 38 Morohongo, Moroyama-machi, Irumagun, Saitama, 350-0451 Japan; 2 Department of Breast Oncology, Saitama Medical University International Medical Center, 1397-1 Yamane, Hidaka, Saitama, 350-1241 Japan; 3 Department of Nuclear Medicine, Saitama Medical University International Medical Center, 1397-1 Yamane, Hidaka, Saitama, 350-1241 Japan; 4 Department of Pathology, Saitama Medical University International Medical Center, 1397-1 Yamane, Hidaka, Saitama, 350-1241 Japan

## Correction

After the publication of this article [1], we noticed that in Fig. 2, the survival curve images (C and D, lower panel) were incorrect. The corrected Fig. 2 is presented below. The correction does not affect in any our results and conclusions.

**Fig. 2:**
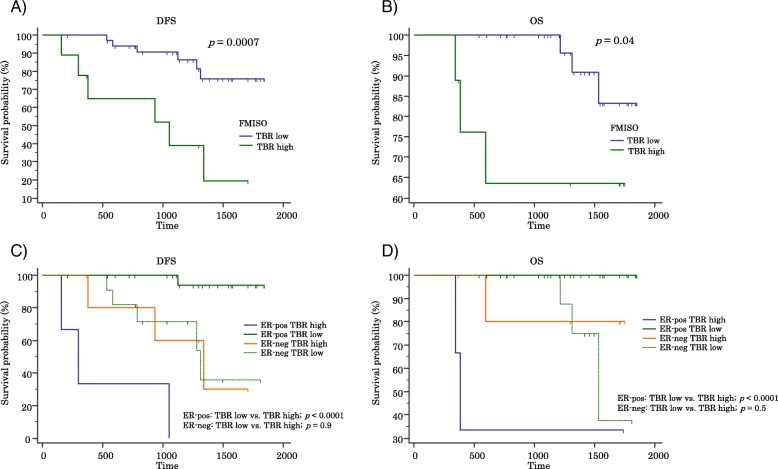
Survival curves. **a** Disease-free survival (DFS). **b** Overall survival (OS). **c** DFS stratified by estrogen receptor (ER) status. **d** OS stratified by ER status. The tentative cutoff value of 1.48 separates tumors with higher ^18^F-fluoromisonidazole tissue-to-blood ratio (TBR high) from those with lower ^18^F-fluoromisonidazole tissue-to-blood ratio (TBR low)
