# Supplementary figures and images for: Intracellular hypoxia measured by 18F-fluoromisonidazole positron emission tomography has prognostic impact in patients with estrogen receptor-positive breast cancer
Source: Breast Cancer Res. 2018 Jul 27;20:78. doi: 10.1186/s13058-018-0970-6 (PMC6063018; doi:10.1186/s13058-018-0970-6)

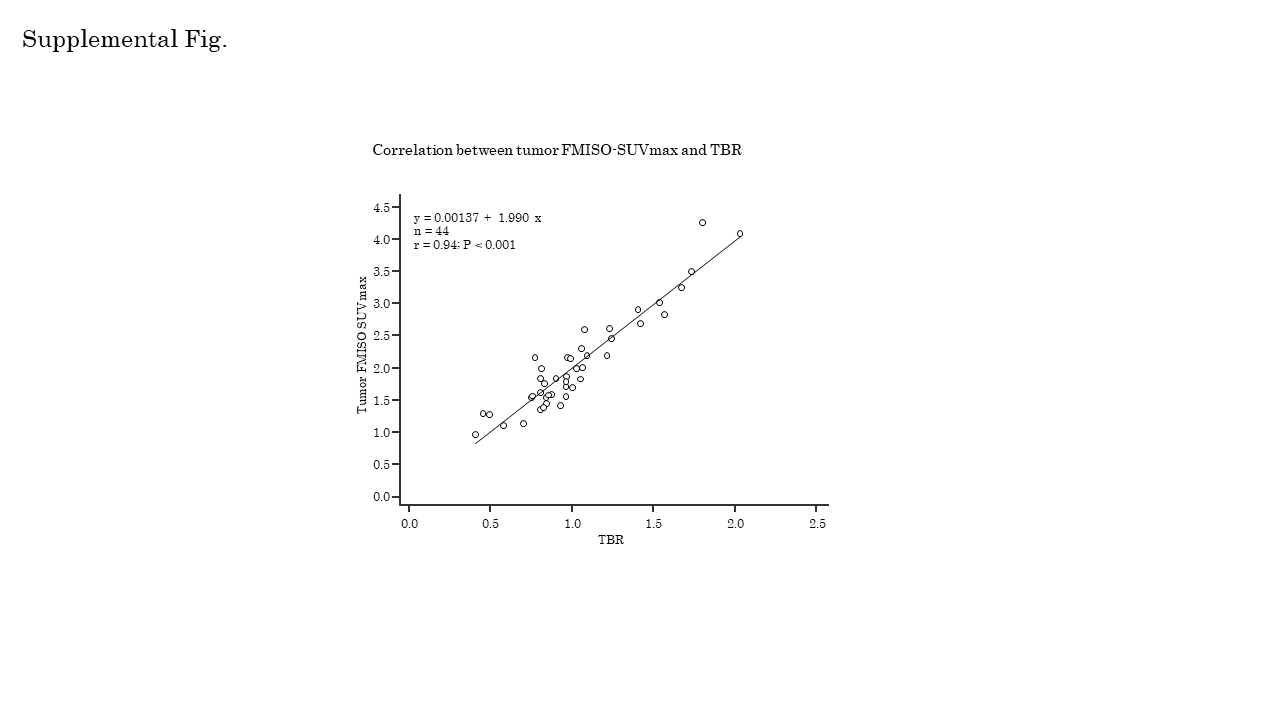

Supplement: Supplementary file 1 — Figure S1. Correlation between tumor FMISO-SUVmax and FMISO-TBR. Regression plots between tumor FMISO-SUVmax and FMISO-TBR showed high correlation at r = 0.94. (TIF 66 kb) [file 13058_2018_970_MOESM1_ESM.tif]
